# Impact of Immuno-Nutrition on the Nutritional Status, Inflammatory Response and Clinical Outcome of Clinic-Admitted Mild-Intensity-COVID-19 Patients: A Pilot, Perspective-Concluding Study

**DOI:** 10.3390/metabo13101070

**Published:** 2023-10-12

**Authors:** Martina Basilico, Marialaura Scarcella, Emanuele Rinninella, Nena Giostra, Stefano Marcelli, Carlo Rasetti, Jan Tack, Ludovico Abenavoli, Emidio Scarpellini

**Affiliations:** 1 Clinical Nutrition Unit and Internal Medicine Unit, “Madonna del Soccorso” General Hospital, Via Luciano Manara 7, 63074 San Benedetto del Tronto, Italy; martinabasilico82@gmail.com (M.B.); nena.giostra@sanita.marche.it (N.G.); carlo.rasetti@sanita.marche.it (C.R.); 2 Anesthesia, Intensive Care and Nutritional Science-Azienda Ospedaliera “Santa Maria”, Via Tristano di Joannuccio, 05100 Terni, Italy; m.scarcella@aospterni.it; 3 Department of Translational Medicine and Surgery, Clinical Nutrition Unit, Catholic University of Sacred Heart, Gemelli Foundation, 00168 Rome, Italy; emanuele.rinninella@unicatt.it; 4 Nurse Teaching Programme Direction, Polytechnics University of Marche, 63036 Ascoli Piceno, Italy; s.marcelli@univpm.it; 5 Translational Research in Gastrointestinal Disorders (T.A.R.G.I.D.), Gasthuisberg University Hospital, KULeuven, Herestraat 49, 3000 Leuven, Belgium; jan.tack@med.kuleuven.be; 6 Department of Health Sciences, University “Magna Graecia”, 88100 Catanzaro, Italy; l.abenavoli@unicz.it

**Keywords:** COVID-19, nutritional status, overweight, immuno-nutrition, inflammation, internal medicine

## Abstract

The SARS-CoV-2 pandemic has impacted our lives since early 2020. Both malnutrition and an overweight status significantly correlate with worse patient outcomes and mortality. Immuno-nutrition (IN) has shown promising results in the inflammatory bowel disease (IBD) clinical course and the extubation time and mortality of patients admitted to intensive care units (ICUs). Thus, we wanted to assess the impact of a standardized IN oral formula on COVID-19 patients admitted to our mild-intensity clinic in late 2021. We prospectively enrolled patients admitted to the Internal Medicine COVID-19 Unit of San Benedetto General Hospital. All patients had biochemical, anthropometric, HRCT chest scan, and nutritional assessments at the time of admission and, after oral immuno-nutrition formula administration, at 15 days of the interval follow up. We enrolled 52 consecutive patients (mean age of 60.9 ± 5.4 years, 17 F, and BMI of 23.5 Kg/m^2^). The main comorbidities were diabetes (20%, type 2: 90%), hyperuricemia (15%), hypertension (38%), chronic ischemic heart disease (12%), COPD (13%), anxiety (10%), and depression (8%). Upon informed consent, 14 patients (mean age of 67.9 ± 5.4 years, 7 F, and BMI of 26.7 Kg/m^2^) were accepted to be administered IN. A moderate to severe overweight status was present in 59% of the patients; MNA test (4.4 ± 0.7) and phase angle (PA) values, suggestive of malnutrition, were present in 13% of the patients. After 15 days of admission, we recorded three deaths (mean age of 68.9 ± 4.1 years, 3 F, and BMI of 27.5 Kg/m^2^). An overweight status significantly correlated with the exitus occurrence (r = 0.65). One death was reported among the IN-treated patients. IN administration was followed by a significant decrease in inflammatory markers with a tendency to be higher than those of non-treated patients. IN prevented the worsening of BMI and PA vs. non-treated patients. In this overweight COVID-19 population, immuno-nutrition prevented malnutrition development with a significant decrease in inflammatory markers.

## 1. Introduction

The treatment of SARS-CoV-2 has differed considerably, and global research has been conducted since January 2020. COVID-19 is the disease caused by this emerging virus and is characterized by interstitial pneumonia and pulmonary thromboembolism. Further, gastrointestinal (GI) and non-GI symptoms are included in COVID-19. The first waves of the pandemic have been found to be harmful to humans [1]. The use of RNA vaccines has changed the clinical features of COVID-19. For example, anosmia and dysgeusia have been replaced with a higher incidence of conjunctivitis [2]. Thus, wave by wave, mortality has fallen by 4–5% and is now lower than seasonal flu [3]. Research performed on the bodies of deceased patients has contributed to the understanding of COVID-19 pathophysiology. It is a hyper-inflammatory syndrome where a non-educated immune system tries to produce antibodies towards a new agent. Subsequently, a cytokine storm is elicited with target organs becoming inflamed, leading to the rapid development of fibrosis [4]. The lungs become “cement” and patients develop subclinical, but terrifying, respiratory failure [1,2].

There is a statistically significant relationship between inflammatory cytokine levels, nutritional status (namely, malnutrition), and COVID-19 mortality [2]. On the other hand, the stage and gravity of obesity significantly correlate with patients’ morbidity and mortality [5]. For example, sarcopenia, typical of obesity, is a risk factor for a worse prognosis of COVID-19 and, also, higher SARS-CoV-2 infection susceptibility [5,6].

The mainstays of COVID-19 treatment are antibiotics, steroids, and low-molecular-weight heparin administered in the second week of infection [7]. There is growing literature evidence on the efficacy of the re-establishment of physiologic immune system functioning via nutritional components, both for the prevention and treatment of COVID-19 [8,9]. Therefore, immuno-nutrition can be defined as the nutrients or specific components of food, in concentrations significantly higher than those of a normal diet, able to modulate the activation of the immune system or the effects due to its activation [10]. Specifically, some immuno-nutrients have been used as add-on treatments of COVID-19 vs. evidence-based standard therapy. Initial studies have shown promising results due to the significant down-regulation of the innate and adaptive immune response involved in the “cytokine storm” typical of COVID-19 patients [11].

Previous data from our study group have already demonstrated the efficacy of a whey-protein-rich enteral feeding formula in ICU-ventilated COVID-19 patients. In detail, we observed an earlier extubation time and improved nutritional status [12]. Further, data from semi-intensive obese COVID-19 patients have confirmed the preventive effect on sarcopenia and malnutrition of the same formula. This result was joined with a significant drop in inflammatory markers and cytokines [13]. In fact, the formula used in former studies by our group is rich in whey proteins and, in particular, in bioactive peptides able to modulate the immune response, especially in hyper-inflammatory diseases like COVID-19 [12,13].

Thus, this prospective exploratory single-center study aims to evaluate the nutritional and anti-inflammatory effects of an outlined nutritional protocol based on immuno-nutrition (IN) in COVID-19 patients admitted to a mild-intensity clinic.

## 2. Materials and Methods

### 2.1. Study Protocol

In this single-center prospective exploratory study, we consecutively enrolled adult COVID-19 patients admitted to the mild-intensity Internal Medicine COVID-19 Unit of “Madonna del Soccorso” General Hospital, San Benedetto del Tronto, Italy, between 1 October and 30 January 2022. We followed regional Ethical Committee regulations for patients’ enrollment (Ethical Committee Marche, Italy). The inclusion criteria were an adult age (namely, higher than 18 years), a confirmed diagnosis of SARS-CoV-2 infection, and no need for mechanical ventilation (both non-invasive/invasive) for at least 48 h. Patients’ treatment followed the accepted updated guidelines for COVID-19 [7].

All patients had a complete medical assessment at the time of admission (T0) and after 15 days following the daily oral administration of an immuno-nutrition (IN) formula (T1); biochemical, anthropometric, high-resolution tomography chest scan (HRCT), and nutritional assessments (namely, MNA test and bioimpedance analysis (BIA)) were also carried out.

The study group was compared with COVID-19 patients not administered an IN formula.

### 2.2. Inclusion and Exclusion Criteria

We enrolled consecutive patients admitted to the mild-intensity Internal Medicine COVID-19 Unit of San Benedetto General Hospital because of SARS-CoV-2 infection.

The exclusion criteria were: pregnancy, artificial nutrition in the previous 15 days before admission, allergy to the immuno-nutrition components, major GI tract surgery, malabsorption syndromes, inflammatory bowel disease, GI motility disorders, acute or chronic pancreatitis, immunodepression (e.g., acquired immunodepression syndrome (HIV)), hematologic disease, and cognitive status impairment.

### 2.3. Immuno-Nutrition Administration Scheme

The immuno-nutrition (IN) formula used in the study is a powdered oral nutritional supplement designed for patients affected by inflammatory bowel disease. In fact, there is much evidence confirming its anti-inflammatory effect, especially in inflammatory bowel disease in children [14].

Its composition consists of: proteins, 3.5 g/100 mL (exclusively comprising casein naturally rich in TGF-ß2); fats, 4.6 g/100 mL (milk fat, MCT, corn oil, soy lecithin, MCT: 25% of total lipids, in order to facilitate rapid replenishment; essential fatty acids equivalent to 4.6% of total calories; limited content of linoleic acid (n-6)); and carbohydrates, 11 g/100 mL (maltodextrin (61%) and sucrose (39%)).

The powder is reconstituted at 20%–1 Kcal/mL: 200 g of powder in 850 mL of water, to reconstitute 1 L of IN formula (1000 Kcal). Later, it is possible to increase the concentration up to 30%–1.5 Kcal/mL: 300 g of powder in 750 mL of water to reconstitute 1 L (1500 Kcal) [14].

The formula was administered once daily (in detail, 300 g of powder in 750 mL of water to reconstitute 1 L (1500 Kcal)) together with the diet of the patient, delivering, on average, 30–40% of the total calories of the daily diet.

### 2.4. Nutritional Assessment

#### 2.4.1. Mini Nutritional Assessment (MNA) Test

The Mini Nutritional Assessment test is a multidimensional screening tool, validated in several clinical settings. More specifically, it is an integrated nutrition index that evaluates different nutritional parameters “to obtain a synthetic information and a more accurate nutritional diagnosis” [15]. MNA has 96% sensitivity, 98% specificity, and 97% predictive value to describe the nutritional status of patients [15].

MNA is useful both for the first and follow-up assessment of the nutritional status of elderly patients [15]. Interestingly, when older patients are hospitalized, MNA scores are able to estimate healthcare costs, length of stay, and short-term and long-term mortality. In fact, the test scores have an inverse correlation with these hospitalization items [15].

More interestingly, the MNA test generates an index of both muscle disability and motility and, in parallel, the nutritional status assessment of hospitalized and non-hospitalized patients [15].

The test is composed of 18 items. These are grouped into three sections: one evaluating anthropometric measurements and weight changes; one estimating the amount and composition of ingested food; and one assessing disabilities and cognitive status [16].

MNA testing encompasses two steps:

- Screening (maximum score of 14, extracted from six variables): Information regarding weight loss over the previous three months, food intake, motility, acute stress, cognitive status, and Body Mass Index (BMI) assessment. In detail, scores between 0 and 7 are predictive of malnutrition, scores between 8 and 11 suggest the risk of malnutrition, and scores between 12 and 14 indicate sufficient nourishment. In particular, for scores lower than 11, it is strongly recommended that the remaining test items continue to be collected. For scores higher than 24, the patient is clearly well nourished. On the other hand, the “grey zone” of scores between 17 and 23.5 suggests a risk of malnutrition. Finally, when patients score less than 17, they are clearly malnourished.
- Self-Global Assessment (namely, evidence of history of drug use, food habits and fluid intake assessment, evaluation of place of residence, and scoring of patient’s considerations of personal health status and nutritional status).

#### 2.4.2. Bioimpedance Analysis

Bioelectrical impedance analysis (BIA) is a non-invasive tool used to assess human body composition (namely, the measurement of fat, bone, water, and muscle content). The BIA device delivers a low-frequency electrical current. The impedance assessment of the body is based on the principle that fluid and cellular structures present different levels of resistance to an electrical current passing through a living system [17]. The BIA device measures: resistance (R—Ohms), estimating cellular hydration; reactance (Xc—Ohms), resembling tissue integrity, and, importantly, phase angle (PA—degrees). The latter is the arc tangent between R and Xc. Thus, BIA can evaluate hydration and nutrition in humans [18].

### 2.5. Data Collection

We prospectively collected anthropometric, clinical, and laboratory test data from the patients’ medical files. In detail, we collected general and demographic variables on the day of admission to the mild-intensity Internal Medicine Unit. All of the other data and parameters measured were recorded daily for the patients’ entire stay, starting from admission to discharge/death. We recorded inflammation and infection markers (CRP, IL-6, white blood cell count and formula, procalcitonin, and erythrocyte sedimentation rate), renal and hepatic function indices, and blood gas analysis variables. The collected data were logged in a database guaranteeing the anonymity of the patients.

### 2.6. Statistical Analysis

Statistical analysis was performed with SPSS Software 21 (IBM, New York, NY, USA). Preliminarily, quantitative variables’ distribution was assessed using the Kolmogorov–Smirnov normality test. All data are presented as mean ± standard deviation (SD) or median (interquartile range [IQR]) according to the normal or not normal distribution. Parametric (Student’s *t*-test) and non-parametric tests (Mann–Whitney U test) were applied to describe the differences between groups for the variables of interest, when appropriate. The alpha level of significance was set at 0.05 [19].

## 3. Results

On 1 October and 30 January 2022, we consecutively admitted 52 consecutive patients (mean age 60.9 ± 5.4 years, 17 females, BMI 23.5 Kg/m^2^) to the mild-intensity COVID-19 Internal Medicine Unit of “Madonna del Soccorso” General Hospital, San Benedetto del Tronto, Italy. Upon informed consent, 14 patients (mean age 67.9 ± 5.4 years, seven females, BMI 26.7 Kg/m^2^) were accepted to be administered with the immuno-nutrition formula.

The patients included in the study had their infection confirmed via SARS-CoV-2 antigenic nasal swab 3.4 ± 0.5 days prior to admission to the Internal Medicine Unit.

Their main comorbidities were: diabetes (20%, type 2 90%), hyperuricemia (15%), hypertension (38%), chronic ischemic heart disease (12%), COPD (13%), anxiety (10%), and depression (8%).

Considering inflammatory markers at enrollment, the median CRP was 29 [5.5–41] mg/L; IL-6 87 pg/mL; white blood cell count 9020 [5900–14,000].

The HRCT scan results were as follows: mild pneumonitis (35%), moderate pulmonary parenchyma involvement (40%), and severe involvement (20%).

The control group (*n* = 18) (COVID-19 patients who did not provide informed consent to receive IN, from the same wave of the pandemic) characteristics are shown in Table 1. The SARS-CoV-2 infection confirmation prior the admission to the Internal Medicine Unit was conducted within a similar timescale to that of the IN-treated group (Student’s *t*-test, *p = NS*).

Of note, the CRP and IL-6 values at the time of Emergency Department admission (namely, before Internal Medicine Unit admission) of both groups did not show significant differences (CRP: 37 ± 1.0 vs. 34 ± 0.9 mg/L for IN and control group, respectively, Student’s *t*-test, *p = NS*; IL-6: 99 ± 2.3 vs. 98 ± 2.1 pg/mL for IN and control group, respectively, Student’s *t*-test, *p = NS*).

**Figure 1:**
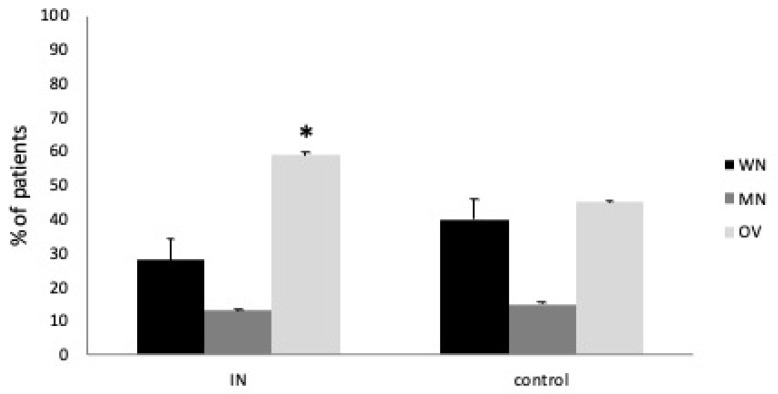
Nutritional status according to MNA test and BIA assessment in IN and control group; WN: well-nourished; MN: malnourished; OV: overweight; Mann–Whitney U test, * *p* < 0.05.

The comorbidity prevalence and other anthropometric, nutritional, and inflammatory characteristics were comparable except for BMI (*p* = 0.05). In addition, the MNA test results and BIA confirmed a statistical difference for overweight representation between the study and control group (Mann–Whitney U test, both *p* < 0.05) (Figure 1).

During their mild-intensity unit stay, all IN and control group patients were treated with guideline-driven treatments (namely, remdesivir, metilprednisolone, piperacillin/tazobactam, and levofloxacin). There was no statistical difference concerning medications used among groups (Student’s *t*-test, *p = NS*).

**Figure 2:**
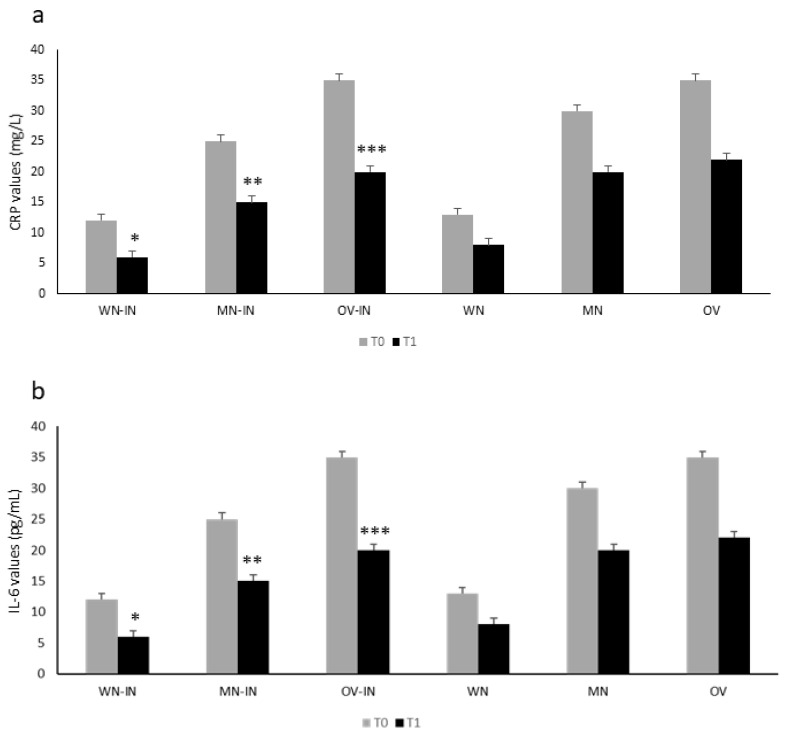
CRP (**a**) and IL-6 (**b**) values according to nutritional status (WN-IN: well-nourished; MN-IN: malnourished; OV-IN: overweight for the IN-treated group; WN, MN, OV for the control group) in the IN and control group at T0 vs. T1. Reduction in inflammatory response markers (IL-6 and CRP) according to nutritional status in the IN group was statistically significant (T0 vs. T1; ANOVA, *, **, ***, all *p* < 0.05). A similar tendency was observed in the control group, without reaching statistical significance (ANOVA, *p = NS*).

Figure 2 shows the inflammatory marker values in the IN group according to their nutritional status at T0. The control group showed a similar behavior at T0 (Figure 2). In both groups, malnutrition and being overweight were significantly associated with higher CRP (Figure 2a) and IL-6 (Figure 2b) values (Student’s *t*-test, both *p* < 0.05).

At T1, all patients showed improved HRCT pneumonitis findings (from severe to moderate, from moderate/mild to mild/significant resolution), except for seven patients (six of whom passed from moderate to severe COVID-19 pneumonitis).

Therefore, after 15 days of admission (T1), three deaths were recorded (mean age 68.9 ± 4.1 years, three females, BMI 27.5 Kg/m^2^). Overweight significantly correlated with the exitus occurrence (non-parametric Spearman test, r= 0.65).

Specifically, one death was reported for IN-treated patients (mean age 71.1 ± 3.1 years, female sex, BMI 26.6 Kg/m^2^), and two patients were moved to ICU care because of worsening respiratory performance. The latter was associated with worsened HRCT pneumonitis findings and COPD relapse, respectively.

In the control group, at T1, we observed two deaths (mean age 70.2 ± 2.7 years, one female, BMI 23.1 Kg/m^2^) and two patients were moved to ICU care because of worsening respiratory performance. The latter was associated with worsened HRCT pneumonitis findings.

After 2 weeks of IN formula administration, we observed a significant reduction in inflammatory markers (PCR, IL-6; ANOVA, *, **, *** *p* < 0.05 for both) in the IN group (Figure 2 a and b). In the control group, a similar trend was observed, without reaching statistical significance (ANOVA, *p = NS*) (Figure 2).

Glycemic assessment was not affected by IN nutrition (*p = NS*).

**Figure 3:**
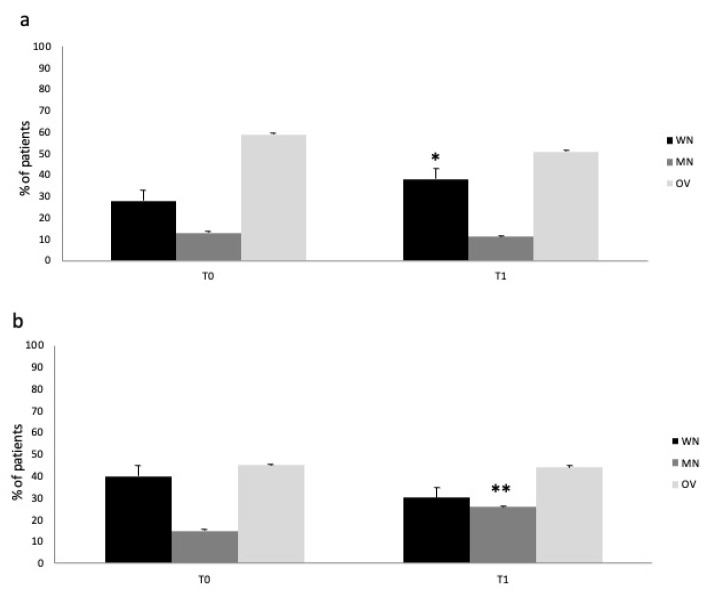
Nutritional status (WN: well-nourished; MN: malnourished; OV: overweight) changes in the IN (**a**) and control group (**b**). IN significantly prevented worsening of nutritional status (with a significant reduction in the percentage of malnourished patients (MN and OV)), ANOVA, * *p* < 0.05. Malnutrition prevalence (MN patients’ percentage) significantly increased in the control group, ANOVA, ** *p* < 0.05.

Figure 3 describes the nutritional status change in the IN and control group from T0 to T1. Immuno-nutrition administration was able to prevent the worsening of nutritional status in the treatment vs. control group (ANOVA, *p* < 0.05) (IN group: Figure 3a; control group: Figure 3b). The finding was due to the significant reduction in malnourished patients (namely, MN and OV) and increase in WN patients in the IN-treated group, respectively (T1: MNA test, percentage of WN, MN, OV patients: 38/11/51%; BIA evaluation PA values for WN, MN, OV patients: 4.6/3.2/8.1°).

Internal Medicine Unit length of stay was not affected by IN use (Student’s *t*-test, *p = NS*).

## 4. Discussion

In this single-center prospective pilot study, COVID-19 patients admitted to the mild-intensity Internal Medicine care clinic of our hospital were treated with immuno-nutrition. Its impact on nutritional status and inflammatory state were evaluated. The control group was represented by COVID-19 patients not administered the IN formula.

In agreement with previous results from our research group [12,13], we have shown that a specific casein-rich immuno-nutrition formula is able to prevent the worsening of nutritional status in prevalently obese COVID-19 patients (namely, a reduction in the percentage of MN and OV patients). This was followed, in parallel, by an inflammatory response reduction. On the other hand, there was a non-significant reduction in inflammatory marker concentrations in the control group. Indeed, malnutrition was not prevented in this subset of individuals infected with SARS-CoV-2.

This study has been the conclusion of our project exploring the nutritional and immunomodulatory potential of a whey-protein-rich formula that showed promising results in terms of reduced inflammatory state in pediatric IBD patients [14]. Starting from ICU patients [12], continuing through semi-intensive ones [13], and concluding with mild-intensity COVID-19 patients, we can report consistent results.

We recorded a significant impact in terms of a reduction in inflammatory markers such as CRP and interleukin-6 in ICU patients. The inflammatory nutritional marker prealbumin followed this trend, demonstrating its acute phase protein origin. The latter reduction was significantly correlated with shorter extubation time and lower mortality rates. Altogether, this anti-inflammatory effect was accompanied by a reduction in malnutrition risk. The IN formula was administrated enterally through a nasogastric tube [12]. Among these patients, there was not a significant percentage of obese subjects.

The subsequent exploratory trial in semi-intensive, but obese, COVID-19 patients from our hospital showed that the orally administered immuno-nutrition was able to significantly reduce the inflammatory response of these sub-acute patients without affecting their mortality rate. Interestingly, this formula was able to prevent both malnutrition worsening and occurrence, although these patients were already suffering from sarcopenia [13].

In the present study, there was no significant correlation between the prevention of malnutrition development and improved mortality or the prevention of worsened clinical course (namely, the need for ICU admission) [12]. This finding can be explained mainly by the small sample size and also by the short follow-up time of the population in the study, which does not allow further speculation on the impact of IN administration on the prognosis of COVID-19 patients.

Interestingly, the reduction in overweight representation from T0 to T1 in both groups did not reach statistical significance, mainly due to the high percentage of obese patients enrolled in both groups and the small sample size. We must also consider the higher prevalence of obese subjects among IN- vs. non-IN-treated patients. This finding may agree with those from semi-intensive COVID-19 patients previously reported from our group [13]. More interestingly, this tendency towards a reduction in OV representation in COVID-19 patients by IN has promising implications for the prognosis, morbidity, and, especially, mortality of SARS-CoV-2-infected subjects. Indeed, the significant relationship between higher mortality and obesity in COVID-19 is well known [6]. In addition, the positive impact of IN on obesity in the general population is also known [20].

We must also consider the differences between the present study and the study on semi-intensive patients enrolled in the previous investigation conducted by our group [13]. In fact, mild-intensity clinic patients are the most represented COVID-19 patients. Although their inflammatory status could be lower than semi-intensive ones, they represent the vast majority of admitted and non-admitted patients. Thus, the impact of IN on their inflammatory status and malnutrition risk and status has important economic implications. For example, the early introduction of IN formulas in the diet of these patients could help reduce the hospital admission rate. This is a crucial point in the emergency setting of a pandemic. In fact, the relatively low cost profile and good compliance of IN administration to SARS-CoV-2 patients is one of the pros in favor of its introduction into the daily clinical management of COVID-19.

Further, IN can guarantee a better prognosis perspective because the reduced risk of malnutrition significantly correlates with improved COVID-19 patient survival rates. On the other hand, semi-intensive ventilated patients can show issues in oral IN formula administration vs. non-ventilated patients.

The findings from the present study are in agreement with the solid evidence showing both the positive impact of nutritional assessment and the use of specific food supplements on COVID-19 patients’ morbidity and mortality [21]. These data are available in both critical and non-critical patients [22,23]. In detail, whey-protein-rich formulas and pre-, pro-, and, lately, postbiotics have been used as add-on treatments for steroid, antibiotic, and antiviral therapy in COVID-19 subjects, with promising results [24,25].

Thus, our study and the previous data on immuno-nutrition in ICU and semi-intensive patients pave the road and endorse the use of nutritional evaluation with validated questionnaires (namely, MNA, MUST) and/or bioimpedance analysis in current clinical practice. This can have a significant impact on patients’ time of hospital stay, morbidity, and, importantly, mortality.

Specifically, we retrieved one Brazilian study evaluating the impact of a protein-rich normo-caloric diet with or without IN formula as an add-on treatment for the inflammatory response (described also by lymphopenia) on non-ventilated COVID-19 patients [26]. In this study, there was a significant reduction in the inflammatory cascade via immuno-nutrition formula administration, but no effect on the nutritional status of the patients. This is an important difference with our investigation and can be explained by the type of immuno-nutrition used and, moreover, by its higher content in proteins. Going deeper, bioactive peptides added to the industrial diet may favor mucosal healing in inflammatory bowel disease with a strong anti-inflammatory effect [27]. These peptides are specific growth factors (namely, transforming growth factor-β (TGF-β)) and control the processes of immune cell differentiation, proliferation, and activation. Thus, these peptides can obtain mucosal immunomodulation with reduced intestinal permeability to inflammatory cytokines, reduced systemic inflammation, and SARS-CoV-2 entrance within the cells [26].

Although a similar trend was observed in the reduction in inflammatory markers in the control group, only the group of patients treated with IN demonstrated a statistically significant reduction in IL-6 and CRP. Thus, these findings support an anti-inflammatory effect of IN. These findings are in line with those from ICU and semi-intensive COVID-19 patients from our group and with several reports from the literature evaluating other IN examples, such as omega-3 fatty acids successfully used in diabetic and septic patients [28]. Furthermore, arginine also showed similar promising results in COVID-19 patients [29].

Our study has several limitations. First, the sample size was small, mainly due to the circumstances of a pandemic and the prospective experimental design. In detail, the small sample size could have affected the lack of significance of the difference in mortality among IN and control groups. Second, our study took into consideration patients enrolled during the third wave of the SARS-CoV-2 pandemic. Their clinical presentation and inflammatory condition differed from those during previous waves (namely, different virus strain, use of vaccine, and antivirals). Third, our cohort had a high representation of obese people with a significant prevalence of sarcopenia that could have benefited from the use of a casein-rich IN formula more than other populations.

## 5. Conclusions

In conclusion, the data from this pilot single-center perspective study showed, for the first time, that a specific casein-rich immuno-nutrition formula was able to prevent malnutrition development in COVID-19 patients admitted to a mild-intensity unit. These results are in agreement with previous data from our research group that also evaluated ICU and semi-intensive COVID-19 subjects. The prevention of malnutrition worsening was significantly correlated with a reduction in the inflammatory response. Finally, the IN formula administration was well tolerated and safe.

To the best of our knowledge, this is the first report using a dedicated IN formula to improve nutritional status and reduce inflammatory storms in COVID-19 patients who do not need mechanical and/or non-invasive ventilation. The relevance and originality of these data can also be easily reproduced and disseminated across the medical board because of the large availability of patients involved in the study, namely, patients not in need of intensive care.

Indeed, larger and multi-centric randomized placebo-controlled studies are needed to confirm these promising results, perhaps in a world more free from the SARS-CoV-2 pandemic.

## Figures and Tables

**Table 1:** Characteristics of the study and control populations at T0.

	COVID-19 pts (*n* = 14)	Control Group (*n* = 18)	*p*-Value
Age (years)	67.9 ± 5.4	66.1 ± 4.5	NS
sex	7 F	10 F	NS
BMI (Kg/m^2^)	26.7 ± 0.5	23.0 ± 0.3	<0.05
Days after SARS-CoV-2 infection enrollment	3.4 ± 0.5	3.5 ± 0.4	NS
MNA test (WN/MN/OV (% of patients))	28/13/59	40/15/45	<0.05
BIA PA (°) (WN/MN/OV)	4.5/3.5/8.2	4.4/3.2/7.4	<0.05
CRP (mg/L)	29 [5.6–31]	24 [6–33]	NS
IL-6 (pg/mL)	87 [35–133]	93 [34–136]	NS
Mild-intensity unit stay (days)	19.1 ± 0.4	19.6 ± 0.5	NS

Table legend: NS: non-significant; F: female sex; MNA: mini nutritional assessment tests’ percentages of patients being: WN: well-nourished, MN: malnourished, OV: overweight; PA: phase angle value obtained through bioimpedance analysis (BIA) measurement, showing agreement with MNA test results; CRP: C-reactive protein; IL-6: interleukin 6.

## Data Availability

Data supporting these results can be found in the patient file database of “Madonna del Soccorso General Hospital”, San Benedetto del Tronto, Italy.
